# Child-Pugh Score and Vitamin D: Exploring a New Frontier in Liver Cirrhosis Assessment

**DOI:** 10.7759/cureus.74738

**Published:** 2024-11-29

**Authors:** Ashish S Munoli, Prakash G Mantur, Vishwanath Malkappa Jalawadi

**Affiliations:** 1 Internal Medicine, Shri. B. M. Patil Medical College Hospital and Research Centre, Vijayapura, IND; 2 Medicine, Shri. B. M. Patil Medical College Hospital and Research Centre, Vijayapura, IND

**Keywords:** anti-inflammatory, liver cirrhosis, prognosis, severity, vitamin d deficiency

## Abstract

This study investigates the relationship between vitamin D levels and liver cirrhosis severity, a leading cause of global morbidity and mortality. Chronic liver diseases, stemming from conditions such as hepatitis, alcohol use, non-alcoholic fatty liver disease, autoimmune diseases, and cryptogenic disorders, disrupt vitamin D metabolism, as the liver converts dietary and skin-derived vitamin D into 25-hydroxyvitamin D (25[OH]D), the primary circulating form. The cross-sectional study conducted at the Department of General Medicine of BLDE (DU) Shri. B. M. Patil Medical College Hospital and Research Centre, Vijayapura, from August 2022 to May 2024, involved 89 patients. Based on the Child-Pugh scoring system, these patients were classified into three classes: class A (good hepatic function), class B (moderate dysfunction), and class C (advanced dysfunction). The study found a significant negative correlation (Pearson r=-0.462, p<0.0001) between vitamin D levels and Child-Pugh scores, indicating that as cirrhosis severity worsens, vitamin D levels decrease. The findings highlight vitamin D deficiency as a marker of disease severity, linking it to increased morbidity and mortality and underscoring its potential as a prognostic tool in managing liver cirrhosis.

## Introduction

In a clinical setting, chronic liver disease (CLD) refers to a liver disease process that causes fibrosis and cirrhosis through a process of gradual liver parenchymal destruction and regeneration. A liver disease known as CLD lasts over six months [1]. CLD’s terminal manifestation is cirrhosis. As the compensatory period progresses into the decompensation stage, several problems arise, and the outlook for survival is considerably lowered [2]. Cirrhotic patients exhibit several nutritional abnormalities, which are associated with disease severity and progression [3]. The alteration in liver synthetic function, altered metabolism, and altered nutritional absorption are thought to be the causes of the symptoms. Worsening of these changes is also linked to increased disease severity and progression [3]. One of the top 10 diseases that kill people in India is hepatic disease. As to the WHO data released in May 2017, the number of fatalities in our nation attributed to liver disorders has reached 259,749, which accounts for 2.95% of all deaths [4].

Ethanol metabolism generates acetaldehyde, inducing oxidative damage and impairing liver function. Diagnosis relies on alcohol history and presents with symptoms ranging from abdominal discomfort to specific complications such as ascites. Hormonal imbalances are common and reversible upon alcohol cessation [5].

The diagnosis of liver cirrhosis and its sequelae can be made quickly, easily, repeatedly, and effectively with ultrasound. It is also noninvasive. When used as a diagnostic tool, ultrasonography has a good sensitivity and is comparable to other investigations [6].

When exposed to ultraviolet B radiation from sunlight or when vitamin D is taken orally through food and supplements, the skin’s epidermal layer synthesizes vitamin D. The first metabolite of vitamin D is 25-hydroxyvitamin D (25[OH]D). The vitamin is further hydroxylated in the kidneys to produce 1,25-dihydroxyvitamin D, which is the active form of the vitamin. Activated vitamin D functions more like a hormone than a specific vitamin due to its pleiotropic properties [7].

The synthetic steroid 25(OH)D regulates the metabolism and absorption of calcium, magnesium, and phosphate and dissolves in fat. In addition to these uses, it has been linked to several additional biological outcomes, including serving as an immunomodulatory and anti-inflammatory drug [8]. In addition to controlling cell division and proliferation, 25(OH)D also possesses anti-inflammatory, antifibrotic, and immunomodulatory effects. These consequences influence the etiology and management of numerous CLD causes [9].

Low vitamin D levels are associated with an increased risk of various complications, including higher mortality rates, bacterial infections, complications from elevated portal vein pressure, and greater severity of fibrosis. Severe liver disease is linked to decreasing albumin, vitamin D binding protein, and vitamin D hydroxylation, all of which are correlated with decreased status of vitamin D [10]. Vitamin D deficiency in cirrhosis of the liver is a result of a number of factors, such as inadequate sun exposure, an imbalanced diet, the use of steroids, reduced absorption of vitamin D from swelling of intestinal cells due to portal hypertension or bile salt disruption from cholestasis, and impaired vitamin synthesis in the skin due to jaundice [11].

The Child-Pugh scoring system is used to clinically stage cirrhosis. There are three categories in this system. A denotes normal liver function, B somewhat compromised liver function, and C denotes severe liver impairment. The five criteria used to grade the patients into the categories above were serum bilirubin, serum albumin, ascites, neurological condition, and prothrombin time [12].

It is essential to weigh the effects of vitamin D in the setting of cirrhosis to inform clinical decisions and provide a basis for creating vitamin D recommendations for cirrhotic patients. This study examined the relationship between a liver cirrhosis patient’s vitamin D levels and the Child-Pugh scoring system.

## Materials and methods

Vitamin D and Child-Pugh scores were evaluated to weigh the severity of the disease in liver cirrhosis patients. The “Department of General Medicine of BLDE (DU) Shri. B. M. Patil Medical College Hospital and Research Center, Vijayapura” conducted this cross-sectional study from August 2022 to May 2024. After the patients were adequately told about the design of the study, informed written consent was obtained using the procedures established by the Institute’s ethics committee. The study evaluated the vitamin D levels among individuals who had liver cirrhosis. A sample size of 89 patients was selected from those admitted to “BLDE (DU) Shri. B. M. Patil Medical College Hospital and Research Center.” The T-distribution sample size estimation approach states that 89 samples would be needed for the study to find a mean with a 95% confidence interval and a precision of 0.9.

Inclusion and exclusion criteria: All patients admitted with a diagnosis of liver cirrhosis, confirmed by abdominal ultrasound, were included in the study. Patients with a history of diabetes mellitus, myocardial infarction, cancer, or chronic kidney disease were excluded from the study. Additionally, patients currently receiving therapy with vitamin D3 supplements were also excluded.

Methods

Patients were selected randomly as they visited our hospital and were examined clinically for all signs of liver disease. Detailed history, especially the amount of alcohol intake in grams/day and the duration of alcohol consumption in years, was obtained, as many patients had developed liver cirrhosis secondary to alcohol consumption. Appropriate medication history was also obtained to omit patients taking medications that affect vitamin D levels.

Tests such as international normalization ratio (INR), 25(OH)D, liver function, renal function, and total blood count were performed on blood samples taken from the patients. An enzyme-linked fluorescence test method was performed to assess the levels of vitamin D using a Beckman Coulter kit. Samples were collected immediately after the clinical examination and within 24 hours of admission. Based on the levels of serum vitamin D (25[OH]D), the patients were classified into four groups: deficient, insufficient, sufficient <20 ng/mL, 20-29.9 ng/mL, 30-100 ng/mL, respectively, and probable toxicity >100 ng/mL. 

Hepatitis B and C status were evaluated to identify the cause of CLD. An abdominal ultrasound was done on each subject. The Child-Pugh score for each patient was determined and categorized into three classes: A (good hepatic function), B (moderately impaired hepatic function), and C (advanced hepatic dysfunction). 

The criteria used to categorize patients based on the Child-Pugh score are as follows: Encephalopathy is scored as None = 1 point, Grades 1 and 2 = 2 points, and Grades 3 and 4 = 3 points; Ascites is scored as None = 1 point, Slight = 2 points, and Moderate = 3 points; Bilirubin levels are scored as under 2 mg/mL = 1 point, 2 to 3 mg/mL = 2 points, and over 3 mg/mL = 3 points; Albumin levels are scored as greater than 3.5 mg/mL = 1 point, 2.8 to 3.5 mg/mL = 2 points, and less than 2.8 mg/mL = 3 points; and INR is scored as under 1.7 = 1 point, 1.7 to 2.2 = 2 points, and above 2.2 = 3 points. Based on the total score, the severity of cirrhosis is classified as Child-Pugh A (5 to 6 points, indicating good hepatic function), Child-Pugh B (7 to 9 points, indicating moderately impaired hepatic function), and Child-Pugh C (10 to 15 points, indicating advanced hepatic dysfunction).

Statistical analysis 

The Microsoft Excel spreadsheet was used to enter data, and SPSS version 20.0 (IBM Corp., Armonk, NY) was utilized for analysis. We calculated means and SDs for continuously distributed variables with a normal distribution and expressed categorical data as frequencies and percentages. The statistical method, such as the Spearman correlation coefficient, used a scattered diagram to determine the link between the continuous variables. For the association between two variables to be called significant statistically, the p-value should be less than 0.05.

## Results

Table 1 provides an overview of the demographic and clinical characteristics of the 89 participants in the study. Of the total, 86 (97%) were male and 3 (3%) were female. The average age of male participants was 46.88±13.18 years, while for females, it was 57.33±7.10 years. Among the male patients, 59 (69%) had a history of CLD compared to 2 (66%) of the female patients. The age distribution revealed that three males were in the under-30 age group, while three were in the over-80 age group. For females, two were in the 50-59 age group, and one was in the 60-69 age group.

**Table 1:** Age and sex distribution of patients with liver cirrhosis

Age (years)	No. of patients	Male	Female
<30	3	3	0
30-39	26	26	0
40-49	27	27	0
50-59	20	18	2
60-69	7	6	1
70-79	3	3	0
80+	3	3	0
Total	89	86	3

Of the 89 patients, 86 (97%) were diagnosed with liver cirrhosis primarily due to alcohol consumption. Hepatitis B was identified as the etiology in 2 (2%) patients, and hepatitis C was the cause in 1 (1%) patient.

Table 2 shows the distribution of clinical symptoms among the study participants. The most common symptoms were abdominal distention and lower limb swelling, which were present in 71.7% of patients (n=64). In contrast, the least reported symptom was altered sensorium, observed in only 2.2% of patients (n=2).

**Table 2:** Spotlight on symptoms: Frequency distribution in liver cirrhosis

Symptoms	Frequency	Percent
Abdomen distension	15	16.9%
Abdomen distension, altered sensorium	3	3.5%
Abdomen distension, black color stools	3	3.5%
Abdomen distension, lower limb swelling	64	71.7%
Abdomen distension, lower limb swelling, black color stools, altered sensorium	2	2.2%
Altered sensorium	2	2.2%
Total	89	100.0%

**Figure 1:**
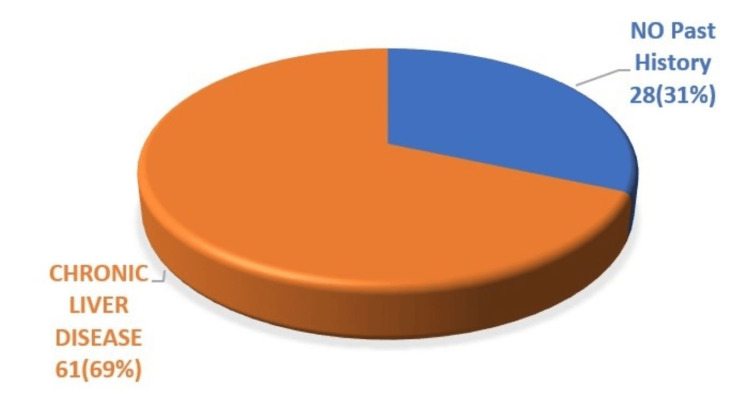
Past history of chronic liver disease

Figure 1 illustrates the distribution of study participants based on their diagnosis history. Out of the 89 patients, 61 (68.5%) had a previous diagnosis of liver cirrhosis, while 28 (31.5%) were newly diagnosed with the condition.

Table 3 presents the alcohol consumption patterns and viral etiology among the 89 patients enrolled in the study. Of the 86 patients who consumed alcohol, 74 (86%) reported consuming more than 150 grams/day, while 12 patients consumed less than 150 grams/day, with an average consumption period of 10 years. Additionally, two patients were diagnosed with hepatitis B and one with hepatitis C, identifying these as the viral etiologies for their liver cirrhosis. 

**Table 3:** Alcohol consumption in patients with liver cirrhosis

		Frequency	Percentage
Alcohol consumption (grams/day)	<150	12	14%
	150+	74	86%
	Total	86	100%
Viral etiology (hepatitis B and C infections)	No alcohol intake	3	
Total		89	

**Figure 2:**
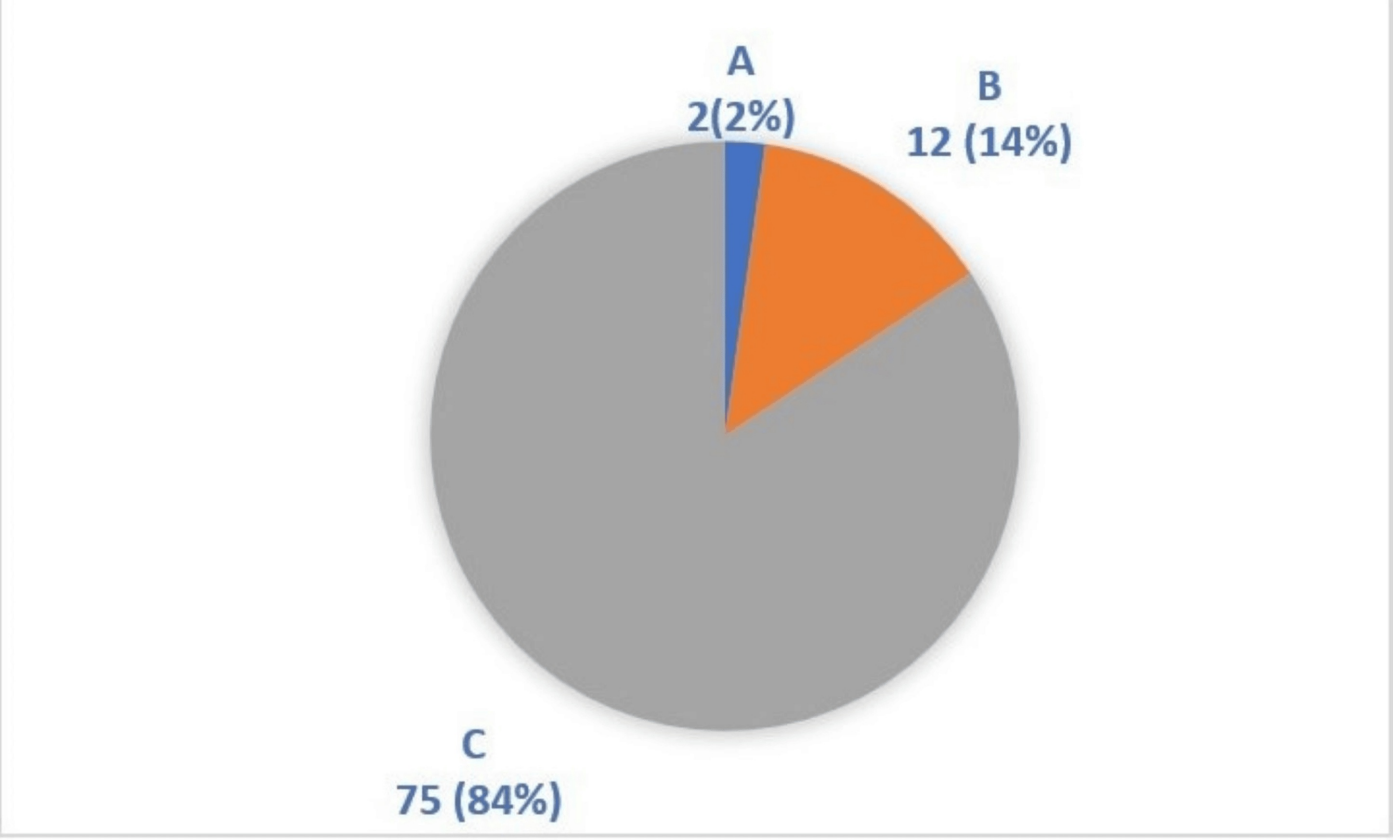
Percentage distribution of patients with liver cirrhosis based on Child-Pugh scores

Figure 2 illustrates the severity of liver cirrhosis in patients based on the Child-Pugh scoring system. The majority of patients (84%, n=75) were classified as Class C, indicating advanced hepatic dysfunction. A small proportion of patients (2%, n=2) were classified as Class A, representing those with good hepatic function.

Table 4 shows the distribution of vitamin D levels among the 89 patients enrolled in the study. The majority of patients (57.3%, n=51) had deficient vitamin D levels (<20 ng/mL), while only a small proportion (3.4%, n=3) had sufficient levels (>30 ng/mL).

**Table 4:** Distribution of vitamin D levels in patients with liver cirrhosis

Levels of vitamin D (ng/mL)	Frequency	Percentage
<20.00 (deficient)	51	57.3%
20.00-29.99 (insufficient)	35	39.3%
30.00 (sufficient)	3	3.4%
Total	89	100.0%

Table 5 shows the distribution of vitamin D levels among the study participants. A total of six patients had low vitamin D levels, consisting of four males and two females, with a mean age of 52±17.39 years.

**Table 5:** Age and etiological distribution of vitamin D levels in patients with liver cirrhosis

Vitamin D level				
	<10 ng/mL	10-20 ng/mL	21-30 ng/mL	>30 ng/mL
No. of patients (n=89) (%)	6(7%)	47(53%)	33(37%)	3(3%)
Sex				
Male (n=86)	4(5%)	46(54%)	33(38%)	3(3%)
Female (n=3)	2(67%)	1(33%)	0	0
Mean age	52±17.39	45.83±11.98	46.06±13.19	61.66±18
Age (years)				
18-35 (n=19)	1	11	7	0
36-55 (n=46)	3	26	16	1
>55 (n=24)	2	10	10	2
Total	6	47	33	3
Etiology of CLD				
Alcoholic (n=86) (%)	4(5%)	46(54%)	33(38%)	3(3%)

Table 6 illustrates the distribution of Child-Pugh class and corresponding vitamin D levels among the study participants. The majority of patients in Class C (29 patients) had reduced vitamin D levels, indicating a potential correlation between more severe liver dysfunction and lower vitamin D status.

**Table 6:** Distribution of vitamin D levels with respect to Child-Pugh class

Child-Pugh class	Deficient levels of vitamin D	Insufficient levels of vitamin D	Sufficient levels of vitamin D
A	0	1	3
B	0	10	2
C	29	36	8

Out of 89 enrolled patients, 90% (80) were discharged and 10% (9) succumbed to death.

**Figure 3:**
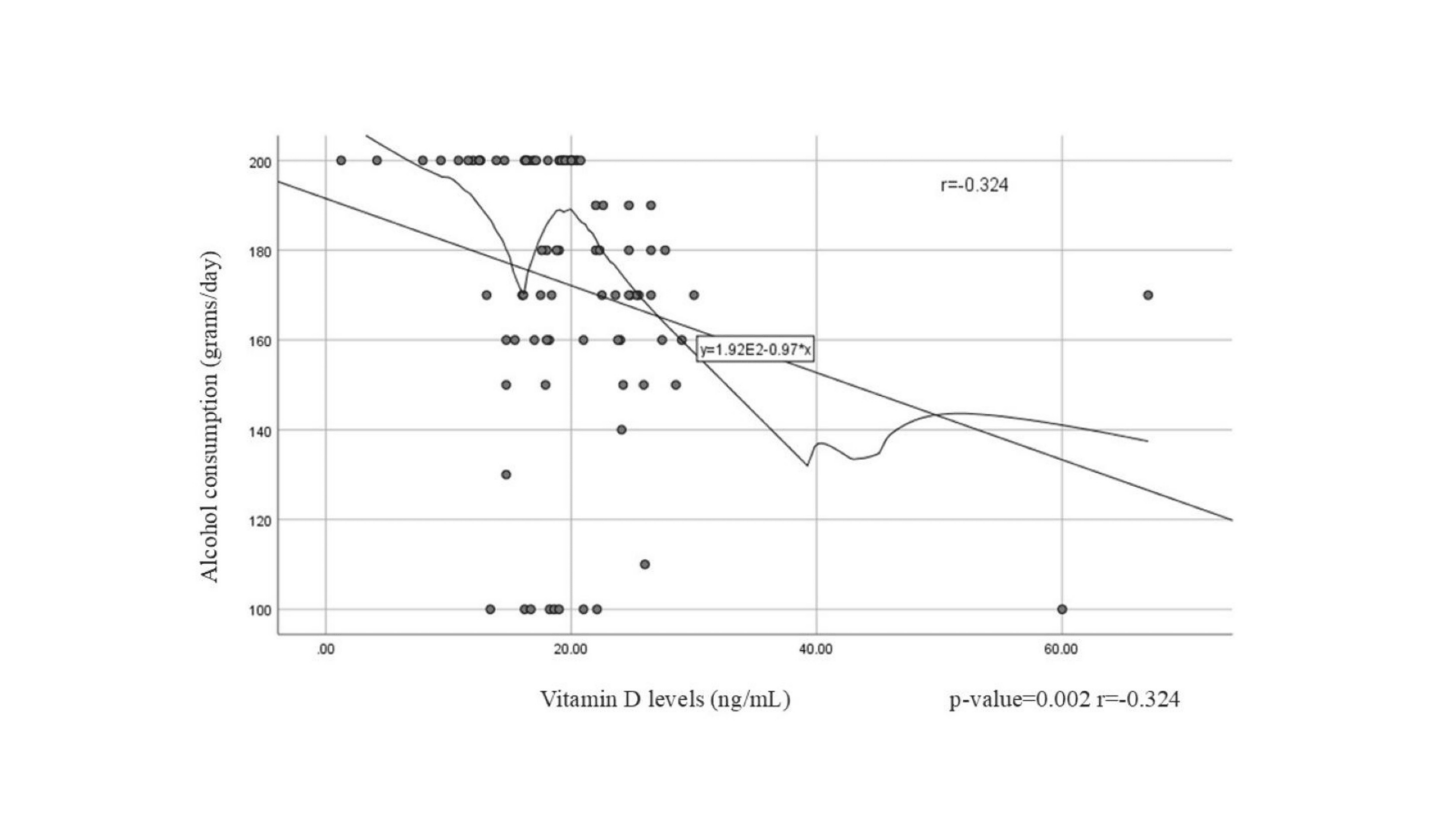
Correlation between vitamin D levels and alcohol consumption in patients with liver cirrhosis

Figure 3 illustrates the relationship between vitamin D levels and alcohol consumption (in grams per day). The X-axis represents vitamin D levels, ranging from 0 to 60 ng/mL in increments of 20, while the Y-axis represents alcohol consumption, ranging from 100 to 200 grams per day in increments of 20. The fitted linear model suggests that for every unit decrease in vitamin D levels, there is a corresponding 0.97 grams per day increase in alcohol consumption, indicating that higher vitamin D levels are associated with lower alcohol consumption. The correlation coefficient indicates an inverse relationship, meaning that as alcohol consumption increases, vitamin D levels tend to decrease.

**Figure 4:**
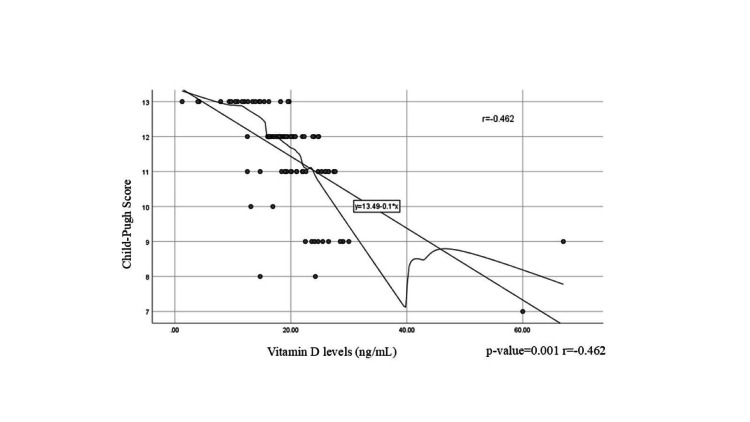
Correlation between vitamin D levels and Child-Pugh score in patients with liver cirrhosis

Figure 4 illustrates the inverse relationship between vitamin D levels and liver disease severity, as assessed by the Child-Pugh score. The X-axis represents vitamin D levels, ranging from 0 to 60 ng/mL in increments of 20, while the Y-axis indicates the Child-Pugh score, ranging from 7 to 13 in increments of 1. The fitted linear model suggests that for each unit decrease in vitamin D levels, there is a corresponding 0.1 unit increase in the Child-Pugh score, indicating that lower vitamin D levels are associated with more severe liver disease. The correlation coefficient demonstrates a negative correlation, meaning that as the Child-Pugh score increases (indicating worsening liver function), vitamin D levels decrease.

## Discussion

The two primary risk factors for CLD, one of the leading causes of morbidity and mortality globally, are widely acknowledged to be alcohol consumption and viral infections. However, according to recent research, CLD appears to be caused mainly by non-alcoholic fatty liver disease in several ethnic groups. Renowned for its function in maintaining bone health and controlling calcium levels, vitamin D also affects cellular functions such as inflammation, apoptosis, and proliferation. Decreased vitamin D levels are linked to liver dysfunction and are correlated with systemic inflammation and the worsening of liver illness.

A total of 89 individuals were enrolled in the study, with 86 males (97%) and 3 females (3%). The mean age for male patients was 46.88 ± 13.18 years, while for females, it was 57.33 ± 7.10 years. Among the male patients, 69% had a prior history of CLD, while 66% of female patients had experienced CLD. In terms of age distribution, 19 men were in the 18-35 age range, 45 men and 1 woman were in the 36-55 range, and 22 men and 2 women were aged 55 and above. This distribution is consistent with the study by Nilajkar et al. [13], which also reported a predominance of male participants (96%) and found a negative correlation between vitamin D levels and Child-Pugh scores.

Out of 89 subjects enrolled in the study, the major cause of liver cirrhosis was the consumption of alcohol, which was consistent with the study carried out by Sharma et al. [14].

Of the 86 patients with alcoholism, 74 (86%) consumed more than 150 grams per day, while 12 (14%) consumed less than 150 grams per day. In that order, the average vitamin D concentrations were 19.57± 8.03 and 22.43± 10.66. The correlation coefficient suggests a negative correlation between vitamin D levels and alcohol consumption. This implies that as vitamin D levels decrease, alcohol consumption tends to increase. This shows that vitamin D levels and alcohol use are inversely correlated, emphasizing the possible necessity for vitamin D status monitoring and management in individuals who consume large amounts of alcohol. This was in line with research that Verma et al. [1] conducted on 100 individuals.

The average level of vitamin D was 42.11±25.30 with Child-Pugh Class A (n=2), 28.63±12.73 with Child-Pugh Class B (n=12), and 17.70±5.32 with Child-Pugh Class C (n=75). Vitamin D levels and the Child-Pugh score have a high negative correlation (r=-0.462, p<0.0001), which suggests that vitamin D levels tend to decrease as the severity of cirrhosis increases. Vitamin D levels and the Child-Pugh score have an inverse relationship, as shown by the correlation value of r=-0.462. According to the Child-Pugh score, reduced vitamin D levels are associated with greater liver disease severity. Patients with CLD may benefit from maintaining higher vitamin D levels to support liver health. This was consistent with the study conducted by Saeki et al. [15].

Of the 89 patients, 80 (90%) were discharged, and 9 (10%) died. The average vitamin D level in discharged patients was 22.38±16.59, while in patients who passed away, it was 10.98±7.04. According to research by Finkelmeier et al. on 251 patients, a deficit (<10 ng/mL) was associated with an increased risk of mortality in patients with liver cirrhosis compared to noncirrhotic patients [16].

A possible correlation exists between reduced status of vitamin D and increased mortality. Compared to discharged patients, those who died had much deficient levels of vitamin D. However, this research suggests that a vitamin D deficit may be associated with less favorable results. An unpaired t-test revealed that the variation in the average vitamin D levels was statistically significant (p=0.0011, p<0.05). This was in line with research done by Patel et al. [17]. 

Kim et al.’s study of 155 patients found that the Model for End-Stage Liver Disease score, Child-Pugh score, and severe vitamin D insufficiency revealed considerably more reliable predictors for short- and long-term patient mortality [18].

Around 345 participants participated in a study by Zhao et al. According to the Child-Pugh score, individuals with liver cirrhosis of varying severity had a significant prevalence of vitamin D insufficiency according to our findings [19].

In a study by Jamil Z et al., 135 participants participated. According to the Child-Pugh score, individuals with liver cirrhosis of varying severity had a significant prevalence of vitamin D insufficiency, which aligns with our findings [20].

The strengths of this study include a comprehensive data collection process that involved detailed patient histories, clinical examinations, and laboratory tests. The use of the well-established Child-Pugh scoring system allowed for an accurate assessment of liver cirrhosis severity. The study also benefits from a relatively large sample size of 89 patients, enhancing its statistical power and reliability. Additionally, the Spearman correlation analysis effectively established a relationship between vitamin D levels and Child-Pugh scores, providing a quantitative measure of the association.

This study has limitations that may impact the generalizability of the findings. First, the exclusion of patients with comorbid conditions such as diabetes mellitus, myocardial infarction, cancer, and chronic kidney disease may limit the applicability of the results to a broader population of patients with liver cirrhosis. These conditions are often present in individuals with liver cirrhosis and are known to be associated with low levels of vitamin D, which could further confound the results. Low vitamin D levels in such patients might lead to falsely low readings, affecting the accuracy of the study’s findings. Therefore, including individuals with these comorbidities would be crucial to providing a more comprehensive and accurate understanding of the interplay between vitamin D and liver cirrhosis in a real-world clinical setting. A large multicentric study is recommended to account for these variables and ensure the broader applicability of the findings.

Second, the study relies on ultrasound imaging for diagnosing liver cirrhosis, which, while widely used, may have variable accuracy based on operator skill and equipment quality. More definitive diagnostic methods, such as liver biopsy or advanced imaging techniques, were not used, which could affect the diagnostic precision.

Additionally, a significant limitation is the lack of a protocol to supplement vitamin D in patients diagnosed with liver cirrhosis and then follow up on their outcomes. Including vitamin D supplementation and monitoring its effects over time could provide valuable insights into its potential impact on liver disease progression and improvement of patient health.

## Conclusions

Our results indicate a negative correlation between Child-Pugh score, a measure of cirrhosis severity, and vitamin D levels, with vitamin D levels decreasing as cirrhosis worsens. While our study suggests that low vitamin D levels are associated with poorer outcomes in liver cirrhosis patients, it is important to note that this is an observational finding. Although significant deficiencies (less than 10 ng/mL) may be linked to higher mortality risks, the causal relationship remains unclear. Given these observations, monitoring vitamin D levels in patients with liver cirrhosis is recommended, particularly for those in advanced stages. Further research is needed to establish whether vitamin D supplementation could serve as an effective adjunct therapy in improving clinical outcomes for cirrhosis patients.

## Figures and Tables

**Figure 5:**
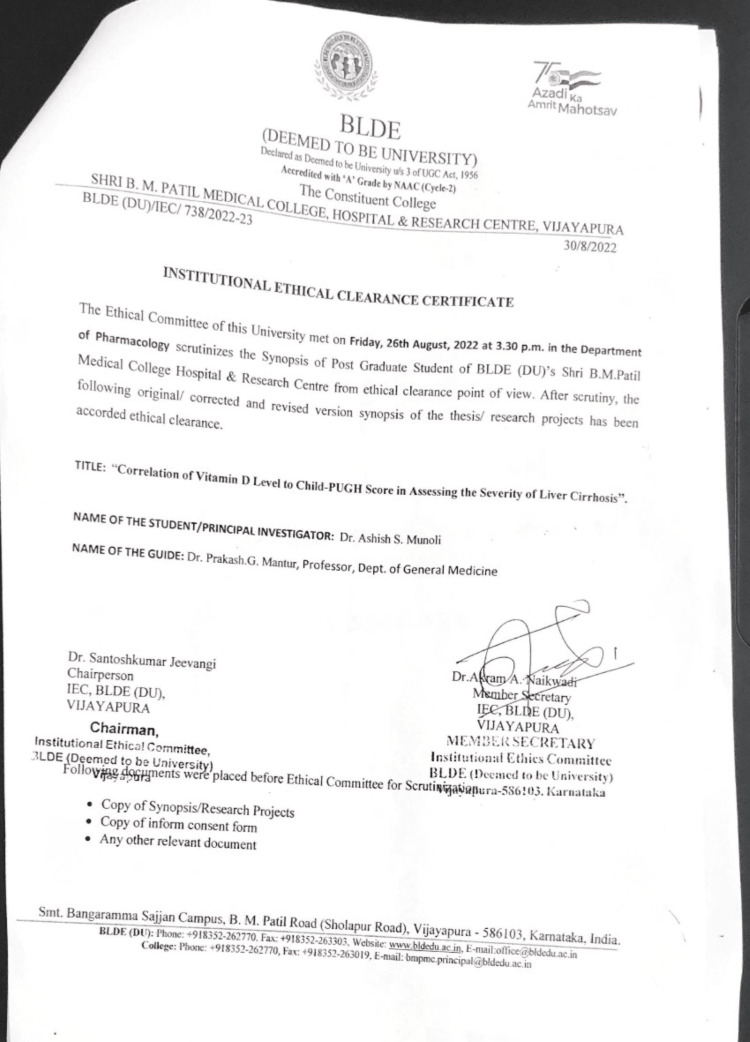
Scanned copy of IRB approval letter IRB, Institutional Review Board

## References

[1] Verma AK, Gautam SK, Giri R (2023). Study of vitamin D level in patients with different etiologies of chronic liver disease and its correlation with Child Pugh class in a tertiary care centre in North India. Int J Res Med Sci.

[2] Yoshiji H, Nagoshi S, Akahane T (2021). Evidence-based clinical practice guidelines for liver cirrhosis 2020. J Gastroenterol.

[3] Kumar P, Chaudhry S, Dev N, Kumar R, Singh G (2020). Serum 25-hydroxyvitamin D level in patients with chronic liver disease and its correlation with hepatic encephalopathy: a cross-sectional study. J Family Med Prim Care.

[4] Mavi K, Kumar A, Singh VK (2021). Estimation of vitamin D level in patients of chronic liver disease and its association with Child Turcotte Pugh's score. Am J Med.

[5] Subramaniyan V, Chakravarthi S, Jegasothy R (2021). Alcohol-associated liver disease: a review on its pathophysiology, diagnosis and drug therapy. Toxicol Rep.

[6] Bhagwat A, Iyer SV (2020). Role of ultrasonography in diagnosis of liver cirrhosis and its complications. Int J Contemp Med Surg Radiol.

[7] Mayr U, Fahrenkrog-Petersen L, Batres-Baires G (2020). Vitamin D deficiency is highly prevalent in critically ill patients and a risk factor for mortality: a prospective observational study comparing noncirrhotic patients and patients with cirrhosis. J Intensive Care Med.

[8] Borella E, Nesher G, Israeli E, Shoenfeld Y (2014). Vitamin D: a new anti-infective agent?. Ann N Y Acad Sci.

[9] Trépo E, Ouziel R, Pradat P (2013). Marked 25-hydroxyvitamin D deficiency is associated with poor prognosis in patients with alcoholic liver disease. J Hepatol.

[10] Khan MA, Dar HA, Baba MA, Shah AH, Singh B, Shiekh NA (2019). Impact of vitamin D status in chronic liver disease. J Clin Exp Hepatol.

[11] Autier P, Boniol M, Pizot C (2014). Vitamin D status and ill health: a systematic review. Lancet Diabetes Endocrinol.

[12] Tsoris A, Marlar CA (2019). Use of the Child Pugh score in liver disease. https://europepmc.org/article/nbk/nbk542308.

[13] Nilajkar GM, Kolwalkar RJ, Prithvi KA (2024). Hepatic decompensation in patients with chronic liver disease: exploring the role of vitamin D deficiency as a prognostic marker. J Assoc Physicians India.

[14] Sharma N, Prakash S, Arora I (2021). Study of vitamin D levels and its correlation with child PUGH score in patients of chronic liver disease. Int J Adv Res Med.

[15] Saeki C, Kanai T, Ueda K (2023). Prognostic significance of sarcopenia and severe vitamin D deficiency in patients with cirrhosis. JGH Open.

[16] Finkelmeier F, Kronenberger B, Zeuzem S, Piiper A, Waidmann O (2015). Low 25-hydroxyvitamin D levels are associated with infections and mortality in patients with cirrhosis. PLoS One.

[17] Patel JK, Mahur HK, Jat SS (2021). A study of the correlation of serum vitamin D levels to Child-Pugh and MELD-Na scoring system in cirrhosis of the liver. Int J Res Med Sci.

[18] Kim TH, Yun SG, Choi J (2020). Differential impact of serum 25-hydroxyvitamin D3 levels on the prognosis of patients with liver cirrhosis according to MELD and Child-Pugh scores. J Korean Med Sci.

[19] Zhao XY, Li J, Wang JH (2016). Vitamin D serum level is associated with Child-Pugh score and metabolic enzyme imbalances, but not viral load in chronic hepatitis B patients. Medicine (Baltimore).

[20] Jamil Z, Arif S, Khan A, Durrani AA, Yaqoob N (2018). Vitamin D deficiency and its relationship with Child-Pugh class in patients with chronic liver disease. J Clin Transl Hepatol.

